# Profiles of economic constraints and marginalization among Korean working adults: differences in work and life outcomes

**DOI:** 10.3389/fpsyg.2025.1563787

**Published:** 2025-05-30

**Authors:** Seran Lee, Hyunjung Yang, Ki-Hak Lee

**Affiliations:** ^1^Department of Psychology, Yonsei University, Seoul, Republic of Korea; ^2^University College, Yonsei University, Seoul, Republic of Korea

**Keywords:** latent profile analysis, psychology of working, economic constraints, marginalization, decent work, life satisfaction

## Abstract

**Introduction:**

Recent research has explored profiles of economic constraints and marginalization based on the Psychology of Working Theory, but such investigations remain limited in Korea.

**Methods:**

This study employed latent profile analysis to identify profiles of Korean working adults based on economic constraints and marginalization and examined their associations with demographic characteristics and job and life outcomes.

**Results:**

Among a sample of 481 Koreans, four distinct profiles emerged: privileged (38.5%), *moderately marginalized* (37.1%), *economically constrained* (14%), and *disadvantaged* (10.4%). The analysis revealed that individuals without a university degree were more likely to belong to the *disadvantaged* and *economically constrained* groups than to the *privileged* group, but no significant gender differences observed. Regarding job and life outcomes, the *privileged* group showed the highest levels of work volition, decent work, physical health, and life satisfaction. The *moderately marginalized* group exhibited lower levels than the *privileged* group. The *economically constrained* group demonstrated similar levels to the *moderately marginalized* group, except that life satisfaction and physical health were comparable to those of the *disadvantaged* group.

**Discussion:**

Based on these findings, theoretical and practical implications are discussed.

## 1 Introduction

Structural issues such as poverty and discrimination represent critical global challenges and are also prevalent in South Korea (Center for Social value Enhancement Studies, 2023). These structural factors have been shown to impact various aspects of life, such as quality of life (Bellani and D’Ambrosio, 2011; Kim and Park, 2015) and career development (Douglass et al., 2017; Duffy et al., 2016). In this context, The Psychology of Working Theory (PWT; Duffy et al., 2016) suggests two structural factors, economic constraints and marginalization, as key factors affecting career development and work experiences. This proposition has been validated extensively by empirical studies conducted across diverse countries and populations (Douglass et al., 2017; Duffy et al., 2024; England et al., 2020).

Despite important findings from previous studies, most have adopted a variable-centered approach that treats populations as homogeneous (Howard and Hoffman, 2018). While variable-centered perspectives describe the relationship between variables, they are limited in providing detailed insights into the characteristics of various subgroups. In contrast, a person-centered approach aims to identify heterogeneous subgroups, thereby facilitating a nuanced understanding of subpopulations (Howard and Hoffman, 2018).

Building on this, recent studies based on PWT have extended the research by adopting a person-centered approach (Duffy et al., 2021; Perez et al., 2023), but no such research has been conducted in Korea. To address this gap, we applied person-centered approach to identify subgroups of structural constraints experiences (economic constraints and marginalization) experienced by Korean working adults. Given the unique Korean cultural context, we expected that a subgroup characterized by higher levels of economic constraints would emerge, which would allow for comparisons of their work and life outcomes with other groups. This, in turn, would enable more tailored recommendations to address the distinct challenges faced by each subgroup.

### 1.1 Economic constraints and marginalization in the PWT

The PWT was developed to shed light on individuals who struggle to freely determine their career due to structural constraints (Duffy et al., 2016). The theoretical model integrates the antecedents and outcomes of decent work, which is defined as work that fulfills economic, relational, and environmental aspects. Decent work is influenced by structural factors—economic constraints and marginalization—and psychological factors—work volition and career adaptability (Duffy et al., 2016). Decent work, in turn, is posited to enhance work and life well-being through satisfaction of individual work needs (Duffy et al., 2016).

Specifically, economic constraints are defined as restricted access to resources, education, and job training due to a person’s or family’s economic situation. Marginalization refers to the perception of being outside the societal mainstream and feeling unrecognized or unimportant, which is influenced by factors such as gender, race, disability, age, and physical appearance (Douglass et al., 2017; Duffy et al., 2016; Gatzweiler and Baumüller, 2014). PWT emphasizes the importance of understanding how individuals’ lifetime experiences of structural constraints influence their career development (Duffy et al., 2024). To capture these experiences, a measure of lifetime economic constraints and marginalization has been developed (Duffy et al., 2019), which reflects individuals’ perceptions of structural barriers across their lives.

### 1.2 Economic constraints and marginalization in the Korean context

The PWT was originally developed within an individualistic cultural framework in the U.S but has been meaningfully applied to collectivist societies such as Korea (Lee and Lee, 2021; Lee et al., 2025). In the Korean context, economic factors are regarded as particularly important. Following its recovery from the International Monetary Fund financial crisis in the late 1990s, Korea achieved remarkable economic growth (Nam and Kim, 2019). However, during this period of growth social disparities also deepened, leading to higher poverty rates and an increased societal emphasis on economic wealth as a core value (Baik and Park, 2004; OECD, 2021). Comparative cultural studies have shown that materialistic values are significantly more pronounced in Korea, with excessive materialism contributing to reduced levels of happiness (Diener et al., 2010a; Koo and Suh, 2015). In this context, economic constraints may have a relatively significant impact on well-being in Korea.

Marginalization is relatively widespread in Korea, as 35% of Korean working adults reported experiencing at least one form of discrimination (Hwang, 2022). These experiences are driven by factors such as gender, educational background, and sexual orientation (Ahn and Jung, 2019). For instance, gender and educational level were identified as significant factors of discrimination (Kim and Williams, 2012), and 5 out of 10 individuals have reported feeling disadvantaged or excluded due to their educational background (Lee, 2023). These findings highlight that marginalization is a pervasive issue in Korea, warranting further investigation of how it intersects with other structural constraints.

In the Korean context, where both economic constraints and marginalization are prevalent, individuals may simultaneously experience these structural challenges, creating compounding disadvantages. This phenomenon can be explained through the lens of intersectionality, which posits that multiple social categories—such as social status, gender, disability, and age—intersect to shape experiences of disadvantage (Collins, 2000; Denise, 2014). Few studies have explored profiles based on experiences of these structural factors. For example, Duffy et al. (2021) identified four distinct profiles among incoming American college students: *Primarily Marginalized*, *Primarily Constrained*, *Privileged*, and *Marginalized and Constrained*. We expected similar profiles to emerge in Korea, while also anticipating the possibility of unique, culture-specific profiles.

### 1.3 Antecedents and outcomes of profile membership

After identifying profiles, we examine whether antecedents and outcomes differed across these profiles. For antecedents, we include gender and educational level, which are closely linked to social vulnerability in Korean society (Kim and Williams, 2012). Korea, for instance, has the largest gender wage gap among OECD countries (OECD, 2022), and women encounter unique career barriers, including gender role expectations (Betz and Fitzgerald, 1987). Regarding educational attainment, approximately half of the individuals reported feeling disadvantaged or excluded due to their educational background, with this tendency more pronounced among those without a university degree (Lee, 2023). Considering this, we expected that women and individuals with lower educational attainment would be more likely to belong to structurally disadvantaged profiles.

For outcomes, we consider key indicators from both work and life domains. Within the work domain, we prioritize work volition and decent work, core constructs within the PWT (Duffy et al., 2016). Work volition defined as the belief that individual can make career decisions when facing external barriers (Duffy et al., 2012). Prior research, including a longitudinal study by Duffy et al. (2020), has demonstrated that economic constraints and marginalization negatively influence work volition and decent work (Douglass et al., 2017; England et al., 2020; Lee and Lee, 2021). Furthermore, marginalization based on educational background has been negatively related to work volition and the perception of achieving decent work among Korean students (Song and Lee, 2023) and working adults (Ahn and Jung, 2019). These studies suggest that structural barriers significantly undermine individuals’ work volition and decent work.

For life outcomes, we include life satisfaction and physical health as key indicators. The PWT suggests that economic constraints and marginalization indirectly influence well-being through decent work (Duffy et al., 2016), though empirical evidence also points to direct effects. Meta-analyses have demonstrated significant associations between subjective social status and well-being (Tan et al., 2020), and between socioeconomic status and physical and mental health (Adler and Ostrove, 1999; Mishra and Carleton, 2015). Marginalization also negatively impacts life satisfaction (Lee and Lee, 2021) and both mental and physical health (Kim and Williams, 2012). These findings suggest that structural barriers also have a detrimental impact on individuals’ overall well-being.

### 1.4 The present study

This study examines subgroups defined by structural constraints among Korean workers and their differences in demographic characteristics and work and life outcomes. We formulated the hypotheses following Spurk et al. (2020). We hypothesize at least four distinct profiles would emerge: (1) low economic constraints and low marginalization (*Hypothesis 1*), (2) high economic constraints and high marginalization (*Hypothesis 2*), (3) high economic constraints and low marginalization (*Hypothesis 3*), and (4) low economic constraints and high marginalization (*Hypothesis 4*). For antecedents, we hypothesize that women and individuals without a university degree are more likely to belong to the high economic constraint and high marginalization profile than to the low economic constraints and marginalization profile (*hypotheses 5* and *6*).

Regarding outcomes, we hypothesize that the low economic constraints and marginalization profile would exhibit the highest work volition and decent work (*Hypotheses 7* and *8*), the high economic constraints and marginalization would exhibit the lowest work volition and decent work (*Hypotheses 9* and *10*). We also hypothesize that profiles characterized by high economic constraints or high marginalization alone will exhibit moderate work volition and decent work—falling between the low–low and high–high profiles (*Hypotheses 11 and 12*). We predict a similar pattern for life satisfaction and physical health that profile with low economic constraints and marginalization would report the highest levels of life satisfaction and physical health (*Hypotheses 13* and *14*), while the group with high economic constraints and marginalization would report the lowest levels (*Hypotheses 15* and *16*). Profiles characterized by high economic constraints or high marginalization alone will exhibit moderate life satisfaction and physical health—falling between the low–low and high–high profiles (*H*ypotheses 17 and 18). Although Duffy et al. (2024) found no significant differences for single-dimension profiles in a U.S. sample, the Korean context warrants an exploratory investigation of outcome differences between these groups (*Research Question 1*).

## 2 Materials and methods

### 2.1 Procedure and participants

The study was approved by the Institutional Review Board at Yonsei University (IRB no. 7001988-202403-HR-1855-04). We recruited Korean working adults aged 19 years and older through Data Spring, an online survey panel provider with 500,000 members stratified by age, gender, and region (about 50% employed). Registered workers received a survey link explaining the study’s purpose, compensation, and confidentiality, and were informed that they could withdraw at any time. Consenting participants completed a 15-min online survey and were compensated with 500 points (about USD 0.38) upon completion. To ensure data quality, we excluded respondents who failed an embedded attention-check item and those with univariate outliers (|z| > 3.29), resulting in the removal of 19 participants (Tabachnick and Fidell, 2013). Harman’s single-factor test indicated that the first factor explained 25.07% of the total variance, below the 50% threshold commonly used to flag common method bias (Podsakoff et al., 2003). Thus, there is little evidence that a single method factor unduly influenced our results.

The final sample comprised 481 Korean working adults (*M* = 41.97 years, *SD* = 10.49), including 51.6% men (*n* = 248) and 48.4% women (*n* = 233). Of the participants, 12.9% (*n* = 62) held a high school diploma, 17.7% (*n* = 85) had an associate degree, 60.3% (*n* = 290) held a bachelor’s degree, and 9.1% (*n* = 44) held a master’s degree or higher. Regarding employment, 80% (*n* = 384) were regular workers, 17.5% (*n* = 81) were temporary workers, and 2.7% (*n* = 13) were classified as others. In addition, 85% (*n* = 409) were full-time workers, 6.9% (*n* = 33) were part-time workers, 4.4% (*n* = 21) were self-employed, 3.5% (*n* = 18) were freelancers. Regarding annual income, 3.3% (*n* = 16) earned less than KRW 20 million, 22.9% (*n* = 110) earned between KRW 20 million and KRW 40 million, 20.2% (*n* = 97) earned between KRW 40 million and KRW 60 million, 27.4% (*n* = 132) earned between KRW 60 million and KRW 80 million, 14.6% (*n* = 70) earned between KRW 80 million and KRW 100 million, and 11.7% (*n* = 56) earned more than KRW 100 million. According to the OECD (2019), incomes of KRW 24 million won (about $17,911) or less are classified as low-income, whereas incomes above KRW 64 million won (approximately $47,877) are classified as high-income.

### 2.2 Measures

#### 2.2.1 Demographic information

Demographic data were collected for the study. Participants responded to questionnaire that included gender, educational level, age, income, employment status, and type of employment.

#### 2.2.2 Economic constraints

We measured economic constraints using the five-item Economic Constraints Scale (Duffy et al., 2019) that assess the economic constraints experienced by participants from the past to the present. Since the scale had not been validated in Korean, we adapted it for our Korean sample. The first author translated the items and two bilinguals back-translated. Equivalence was confirmed through similarity ratings. Participants rated each item on a seven-point Likert scale, ranging from 1 (strongly disagree) to 7 (strongly agree). An example item is, “Through most of my life, I have struggled financially.” Cronbach’s *α* of this scale was 0.94 in Duffy et al.’s (2019) study and 0.87 in this study.

#### 2.2.3 Marginalization

We measured lifetime experience of marginalization using the Lifetime Experiences of Marginalization Scale (Duffy et al., 2019). Because the scale had not been validated in Korean, translated and back-translated the items following the same protocol used for the Economic Constraints Scale. The scale consists of three items that measure the degree of marginalization experienced by participants throughout their lives. Participants rated items on a seven-point Likert scale (1 = strongly disagree to 7 = strongly agree). The following definition of marginalization was provided before answering the items:

We are interested in understanding the extent to which you perceive yourself as marginalized in South Korea. By marginalized, we mean holding a less powerful position in society, being socially excluded, and/or having limited access to resources because of being part of a particular group, possessing a specific identity, or having a certain life history. This often stems from characteristics such as gender, race/ethnicity, sexual orientation, disability status, religious beliefs, physical appearance, or belonging to other minority groups/identities. With this definition in mind, please respond to the following items based on your experiences throughout your life.

An example item is “I have felt marginalized within various community settings for as long as I can remember.” Cronbach’s *α* of this scale was 0.94 in Duffy et al.’s (2019) study and 0.94 in this study.

#### 2.2.4 Work volition

To assess work volition, we used the Korean version of the Work Volition Scale, originally developed by Duffy et al. (2012) and validated for Korean working adults by Kim and Lee (2022). The scale comprises 11 items across three domains: volition, structural constraints, and financial constraints. Participants responded using a seven-point Likert scale, ranging from 1 (strongly disagree) to 7 (strongly agree). Example items include “I feel that I am able to change jobs if I want to” and “Due to my financial situation, I need to take any job I can find” (reverse coded). The Cronbach’s *α* of this scale ranged from 0.77–0.79 in Duffy et al. (2012), 0.74–0.79 in Kim and Lee (2022), and 0.67–0.80 in the present study.

#### 2.2.5 Decent work

To measure decent work, we utilized the Korean version of the Decent Work Scale, which was originally developed by Duffy et al. (2017) and validated for use with Korean working adults by Nam and Kim (2019). The scale comprises 15 items divided into five subscales: safe working conditions, adequate compensation, access to healthcare, free time and rest, and organizational values that align with family and social values. Participants rated the items using a seven-point Likert scale from 1 (strongly disagree) to 7 (strongly agree). Example items include “I feel physically safe interacting with people at work” and “I am not properly paid for my work” (reverse coded). Cronbach’s α of this scale ranged from 0.79–0.97 in Duffy et al. (2017), 0.74–0.94 in Nam and Kim (2019), and 0.79–0.92 in the current study.

#### 2.2.6 Life satisfaction

To assess life satisfaction, we used the Korean version of the Life Satisfaction Scale, originally developed by Diener et al. (1985) and validated for the Korean population by Lim (2012). This scale consists of five items, with participants rating their life satisfaction on a seven-point Likert scale (1 = strongly disagree to 7 = strongly agree). Example items include “So far, I have gotten important things that I want in my life” and “I am satisfied with my life.” Cronbach’s α of this scale was 0.87 in Diener et al. (1985), 0.84–0.91 in Lim (2012), and 0.91 in this study.

#### 2.2.7 Physical health

We assessed subjective physical health using two self-rated items, developed by Frone (2008). Participants responded on five-point scales to the following items: “Over the past month, in general, would you say your physical health is (poor, fair, good, very good, or excellent)?” and “Over the past month, in general, compared to most people your age, is your physical health (much better, somewhat better, about the same, somewhat worse, or much worse)?” The subjective health scale is widely employed in public health and epidemiological research and has been linked to both organizational commitment and mental health. In this study, the two items demonstrated high correlation (*r* = 0.84). In Frone’s (2008) study, the scale was associated with physical and mental health.

### 2.3 Statistical analysis

We conducted LPA using Mplus 8.7 to identify latent subgroups based on participants’ response patterns. Unlike traditional cluster analysis, LPA utilizes a variety of statistical criteria and posterior probabilities to distinguish between profiles (Magidson and Vermunt, 2002). To determine the optimal number of profiles, we considered information indices including Akaike information criterion (Akaike, 1974), Bayesian information criterion (Schwarz, 1978), and sample-size-adjusted Bayesian information criterion (Sclove, 1987), with lower values indicating better model fit. Model comparisons were also evaluated using the Lo–Mendell–Rubin adjusted likelihood ratio test (Lo et al., 2001) and the bootstrapped likelihood ratio test (Peel and McLachlan, 2000), where a significant *p*-value suggested retention of k profiles over k-1. Classification quality was assessed using an entropy value, with scores closer to 1.0 indicating clearer classification; values above 0.80 were considered good (Clark and Muthén, 2009) and values between 0.60 and 0.80 acceptable (Jung and Wickrama, 2008). We also ensured that each profile contained at least 1% of the sample or more than 25 cases (Lubke and Neale, 2006) as well as the interpretability of the profiles.

To examine the relationships between latent profiles, antecedents, and outcomes, we employed the three-step approach (Asparouhov and Muthén, 2014). This approach addresses biases associated with the traditional one-step method by estimating these associations independently. For antecedents, we performed multinomial logistic regression using the R3STEP command in Mplus and the resulting odds ratios elucidating how variations in antecedents corresponded to the likelihood of profile membership (Morin et al., 2016). For outcomes, we used the BCH command to compare differences in work and life-related factors across the profiles and conducted a Wald test with *p*-values adjusted according to Holm’s sequential Bonferroni method (Holm, 1979). Effect sizes for pairwise comparisons were assessed using Cohen’s *d*, with values of 0.2, 0.5, and 0.8 interpreted as small, medium, and large effects, respectively (Cohen, 1988).

## 3 Results

### 3.1 Preliminary analyses

Preliminary analyses were conducted using SPSS 26.0. Results of the descriptive statistics and correlation analysis are presented in Table 1. All variables satisfied the assumptions of normality, with the skewness and kurtosis values for the scales ranging from −0.10 to 0.41 and from −0.61 to 0.42, respectively (Curran et al., 1996).

**Table 1:** Descriptive statistics and bivariate correlations of variables.

	1	2	3	4	5	6
1. Economic constraints						
2. Marginalization	0.42					
3. Work volition	−0.44	−0.33				
4. Decent work	−0.25	−0.27	0.49			
5. Life satisfaction	−0.47	−0.24	0.49	0.46		
6. Physical health	−0.28	−0.23	0.26	0.35	0.52	
*M*	3.83	2.97	3.94	4.39	3.62	2.61
*SD*	1.29	1.40	0.83	0.76	1.28	0.82

*N* = 481. All correlations are significant at the *p* < 0.001 level.

### 3.2 Latent profile analysis

We conducted LPA by sequentially increasing the number of profiles from two to six, with the results presented in Table 2. Entropy values ranged from 0.69 to 0.78 for profiles (Jung and Wickrama, 2008), indicating acceptable classification quality. Information criteria began to level off at four-profile solutions, indicating that this solution may be optimal (Nylund-Gibson and Choi, 2018). Bootstrapped likelihood ratio test showed that the *p*-values became insignificant when moving to a six-profile solution, suggesting that a five-profile solution was statistically superior. We further examined the sample sizes and patterns of the profiles to determine the optimal solution between the four-and five-profile solutions. In the five-profile solution, the added profile contained fewer than 25 cases, which fell below the recommended threshold for profile inclusion (Lubke and Neale, 2006). Moreover, a group in the four-profile solution was divided into two profiles in the five-profile solution, both of which exhibited similar patterns and differed only in magnitude. We selected the more parsimonious four-profile solution given the absence of theoretical distinctiveness and limited interpretive value of the additional profile.

**Table 2:** Latent profile identification fit indices.

	LL	AIC	BIC	SaBIC	LMR (p)	BLRT (p)	Entropy
2-profile	−1642.708	3299.415	3328.862	3306.643	<0.01	<0.01	0.695
3-profile	−1625.725	3271.451	3313.516	3281.776	0.1286	<0.01	0.743
4-profile	−1607.208	3240.417	3295.102	3253.840	0.0613	<0.01	0.730
5-profile	−1595.005	3222.009	3289.314	3238.530	0.0009	<0.01	0.761
6-profile	−1592.292	3222.584	3302.509	3242.202	0.1819	0.50	0.776

LL = log-likelihood; AIC = Akaike Information Criterion; BIC = Bayesian Information; Criterion; SaBIC = sample-size adjusted BIC; LMR = Lo–Mendell–Rubin test; BLRT = Bootstrapped Log-likelihood Ratio Test.

**Figure 1:**
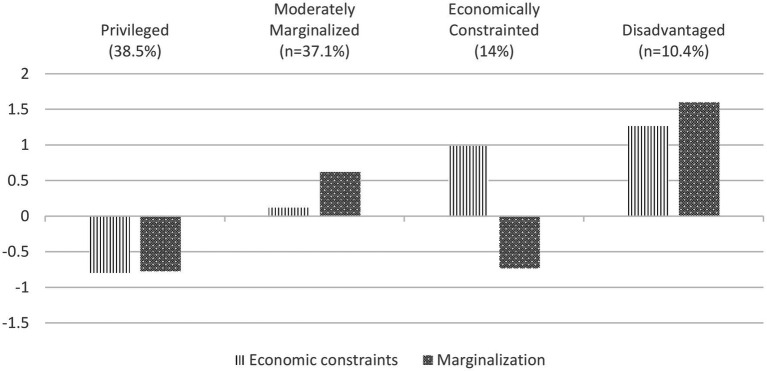
Profiles of economic constraints and lifetime marginalization.

Figure 1 presents the four profiles of economic constraints and marginalization. The first profile, labeled *privileged*, consisted of individuals with low levels of both economic constraints marginalization and represented 38.5% of the total sample. The second profile, labeled *moderately marginalized*, included individuals with moderate levels of marginalization and average levels of economic constraints, and represented 37.1% of the sample. The third profile, labeled *economically constrained*, consisted of individuals with high levels of economic constraint and low levels of marginalization, representing 14% of the sample. The fourth profile, labeled *disadvantaged*, consisted of individuals with high levels of both economic constraints and marginalization, representing 10.4% of the sample. Each profile’s descriptive statistics and mean differences were summarized in Table 3, with differences tested via ANOVA and Scheffé post-hoc comparisons.

**Table 3:** Means, standard deviations, and mean differences for each profile.

Component	Privileged (38.5%)	Moderately marginalized (37.1%)	Economically constrained (14%)	Disadvantaged (10.4%)	Profile group differences
	*M* (*SD*)	*M* (*SD*)	*M* (*SD*)	*M* (*SD*)	
Economic constraints	−0.84 (0.60)	0.12 (0.60)	1.15 (0.53)	1.47 (0.58)	D > C > B > A
Marginalization	−0.81 (0.46)	0.67 (0.48)	−0.77 (0.46)	1.72 (0.55)	D > B > A = C

The mean and standard deviation of each profile were standardized to z-scores.

### 3.3 Profile group differences (gender and educational level)

Associations between profile membership and demographic variables are detailed in Table 4. Individuals without a university degree were more likely to be *economically constrained* (*p* < 0.05) or *disadvantaged* (*p* < 0.01) profiles than the *privileged* profile. No difference was found between the *economically constrained* and *disadvantaged* profiles. Regarding gender, there were no differences except that women were more likely to belong to the *economically constrained* profile than to the *moderately marginalized* profile, which was significant at the *p* = 0.10 level.

**Table 4:** Results of the multinomial logistic regression analysis on antecedent factors.

	1 (ref) vs. 2		1 (ref) vs. 3		1 (ref) vs. 4		2 (ref) vs. 3		2 (ref) vs. 4		3 (ref) vs. 4	
Demographics	Coef. (*SE*)	*OR*	Coef. (*SE*)	*OR*	Coef. (*SE*)	*OR*	Coef. (*SE*)	*OR*	Coef. (*SE*)	*OR*	Coef. *(SE)*	*OR*
Gender	−0.13 (0.27)	1.48	0.63 (0.42)	4.23	−0.13 (0.40)	1.93	0.77^†^ (0.41)	4.83	0.01 (0.45)	2.42	−0.76 (0.49)	1.22
Edu level	0.54^†^ (0.32)	3.23	1.64^***^ (0.42)	11.75	1.35^**^ (0.45)	8.73	1.10^**^ (0.40)	6.57	0.80^†^ (0.46)	5.45	−0.29 (0.48)	1.93

Ref = reference group; Edu level = Educational level; OR = Odds Ratio; Coef. = Coefficient; Gender was coded as 0 = men, 1 = women; Education level was coded as 0 = at or above a university degree, 1 = less than a university degree. Profile 1 = privileged; Profile 2 = moderately marginalized; Profile 3 = economically constrained; Profile 4 = disadvantaged. ^***^p < 0.001, ^**^p < 0.01, ^*^p < 0.05, ^†^*p* < 0.10.

Chi-square goodness-of-fit tests (Table 5) assessed whether each profile’s demographic composition differed from the overall sample. We examined standardized residuals to pinpoint significant deviations between observed and expected counts. For women, no residuals reached significance, indicating that the proportions of men and women did not differ across profiles. By contrast, participants whose highest education was an associate degree or below were significantly underrepresented in the *privileged* profile and overrepresented in the *economically constrained* profile (both *p* < 0.05), confirming their greater likelihood of membership in the latter group.

**Table 5:** Chi-square goodness-of-fit test for the four profile groups.

*N* (%)					
Categorical Variable	Privileged	Moderately marginalized	Economically constrained	Disadvantaged	Total
Women	90 (38.6%)	85 (36.5%)	38 (16.3%)	20 (8.6%)	233 (48.4%)
Individuals without a university degree	40 (27.2%)^*^	56 (38.1%)	31 (21.1%)^**^	20 (13.6%)	147 (30.6%)

Group proportions: Privileged = 38.5%, Moderately marginalized = 37.1%, Economically constrained = 14%, Disadvantaged = 10.4%.

### 3.4 Analysis of outcomes

#### 3.4.1 Work-related outcomes

The results of work and life outcomes are presented in Table 6. Work volition was highest in the *privileged* profile (*M* = 4.39), followed by the *moderately marginalized* (*M* = 3.80), *economically constrained* (*M* = 3.57), and the *disadvantaged* profiles (*M* = 3.27). Effect sizes showed a large difference between the *privileged* and *disadvantaged* profiles (Cohen’s *d* = 1.18), and medium differences between the *privileged* profile and the *moderately marginalized* (*d* = 0.67) and *economically constrained* profiles (*d* = 0.79).

**Table 6:** Mean differences in work-and life-related outcomes across profiles.

Outcome	Privileged (a)	Moderately marginalized (b)	Economic constraints (c)	Disadvantaged (d)	Overall test	Profile differences
	*M (SE)*	*M (SE)*	*M (SE)*	*M (SE)*	*x^2^*	
Work volition	4.39(0.07)	3.80(0.06)	3.57(0.15)	3.27(0.13)	82.037^***^	a > b > d; a > c
Decent work	4.68(0.06)	4.23(0.06)	4.23(0.12)	4.07(0.13)	35.923^***^	a > b = c = d
Safety	5.15(0.08)	4.70(0.09)	5.06(0.17)	4.20(0.22)	27.923^***^	a > b = d; a = c > d
Health	5.36(0.09)	4.62(0.09)	5.25(0.17)	4.89(0.20)	33.344^***^	a > b = d; a = c > b
Compensation	4.15(0.11)	3.75(0.10)	3.34(0.20)	3.57(0.23)	14.658^***^	a > c; a = b = d
Free time	4.60(0.10)	3.99(0.10)	3.84(0.18)	3.79(0.22)	26.513^***^	a > b = c = d
Value	4.28(0.09)	3.99(0.10)	3.64(0.21)	3.84(0.21)	10.562^*^	a > c; a = b = d
Life satisfaction	4.27(0.10)	3.60(0.10)	2.62(0.22)	2.48(0.23)	85.996^***^	a > b > c = d
Physical health	2.91(0.07)	2.56(0.07)	2.27(0.12)	2.01(0.13)	43.069^***^	a > b > c = d

^***^p < 0.001. Safety = Safe working conditions; Health = Healthcare access; Compensation = Adequate compensation; Free time = Free time and rest; Value = Value alignment. Mean differences were assessed using the Wald test with Holm’s sequential Bonferroni-adjusted *p*-values.

Decent work was highest in the *privileged* profile (*M* = 4.68), followed by the *moderately marginalized* (*M* = 4.23), *economically constrained* (*M* = 4.23), and *disadvantaged* profiles (*M* = 4.07). There was no significant difference between the three lower profiles. Effect sizes indicated medium differences among the *privileged* profile and the *disadvantaged* (*d* = 0.72), *moderately marginalized* (*d* = 0.56), and *economically constrained* profiles (*d* = 0.53).

Examining the five decent-work components, the Privileged group scored highest on all dimensions. Notably, safe working conditions (*privileged M* = 5.15 vs. *economically constrained M* = 5.06) and healthcare access (*privileged M = 5.36* vs. *economically constrained M* = 5.25) did not differ between those two profiles. However, the *economically constrained* group scored significantly lower than the *privileged* group on adequate compensation, free time and rest, and value alignment.

#### 3.4.2 Life-related outcomes

Life satisfaction differed significantly across profiles: the *privileged* group scored highest (*M* = 4.27), followed by the *moderately marginalized* group (*M* = 3.60). The *economically constrained* (*M* = 2.62) and the *disadvantaged* (*M* = 2.48) group exhibited the lowest level, with no significant difference between them. Effect sizes indicated large differences between the *privileged* and both the *economically constrained* (*d* = 1.11) and *disadvantaged* profiles (*d* = 1.26). A medium difference was also found between the *moderately marginalized* and *economically constrained* profiles (*d* = 0.67).

Physical health followed the same ordering—Privileged (*M* = 2.91), Moderately Marginalized (*M* = 2.56), Economically Constrained (*M* = 2.27), Disadvantaged (*M* = 2.01)—with no significant difference between the two lowest profiles. Effect sizes indicated a large difference between the *privileged* and *disadvantaged* profiles (*d* = 0.95), and a medium difference between the *privileged* and *economically constrained* profiles (*d* = 0.67).

## 4 Discussion

The present study identified distinct profiles among Korean working adults based on economic constraints and marginalization, revealing both patterns consistent with prior research and unique features shaped by Korea’s cultural and societal context.

### 4.1 Profile membership

Four distinct profiles were identified among Korean working adults, as hypothesized*: privileged*, *moderately marginalized*, *economically constrained*, and *disadvantaged*. This finding parallels the profiles reported by Duffy et al. (2021) among students in the U.S. These findings suggest that individuals’ experiences can be organized along the axes of economic constraints and marginalization across diverse age groups and cultural backgrounds. Such profiles may reflect broader, universal patterns rather than being restricted to specific contexts.

Among the identified profiles, the *privileged* group, characterized by low levels of economic constraints and marginalization, comprised only 38.5% of the total sample. This proportion is notably lower than that reported by Duffy et al. (2021), where more than half of the incoming American students were classified within the privileged group, although direct comparison is challenging due to differences in the sample characteristics. This finding highlights the pervasiveness of these challenges in Korean society. In particular, 10.4% of the sample were classified as experiencing high levels of economical constraints and marginalization, suggesting that approximately one in ten Koreans may belong to this vulnerable group.

The remaining two groups, the *economically constrained* and the *moderately marginalized* group, were characterized by experiencing either significant economic constraints or marginalization, respectively. Interestingly, the *economically constrained* group reported relatively low levels of marginalization, similar to those of the *privileged* group. Although this may seem counterintuitive given the notable difference in economic constraints between the two groups, it parallels findings from a similar study by Duffy et al. (2021), who identified a *“primarily constrained”* profile characterized by high economic constraints and low marginalization among U.S. college students. Prior research has also suggested that while economic constraints and marginalization are moderately correlated, they are conceptually distinct, and individuals may experience one without necessarily experiencing the other (Duffy et al., 2021; England et al., 2020). These findings suggest that even under economic disadvantage, individuals may be protected from experiencing marginalization through access to other resources such as social capital or strong community networks. Lastly, the *moderately marginalized* group represents individuals who reported moderate levels of marginalization and average levels of economic constraints. This group accounted for 37.1% of the sample, underscoring the widespread prevalence of marginalization within Korea. This high proportion is consistent with the National Human Rights Commission of Korea (2023), which reported that 43.7% of Koreans have experienced discrimination in Korean society.

### 4.2 Demographic differences in profile membership

As expected, individuals without a university degree were more likely to belong to the *disadvantaged* or *economically constrained* profiles compared to the *privileged* profile, being 11.75 times more likely to be classified in the *economically constrained* group. This suggests that individuals with lower educational attainment face greater structural barriers within Korean society. These results align with previous research identifying education as a major discrimination source in Korea (Kim and Williams, 2012) and with findings from Statistics Korea (2021) indicating that wage gaps based on educational attainment are widening. At the same time, it is also possible that profile membership itself may have constrained access to higher education. Previous studies have shown that students from lower socioeconomic backgrounds are more likely to experience “undermatching” in college admissions (Cheon and Jeong, 2020). Recent findings also indicate that four-year college enrollment rates are lowest among adolescents who experienced chronic poverty during youth (Hwang and Lee, 2023). These findings suggest that structural factors such as economic disadvantage can significantly restrict access to higher education. Therefore, the relationship between educational attainment and profile membership may be reciprocal rather than unidirectional.

Contrary to expectations, the analysis revealed no significant gender differences, suggesting gender alone may not fully determine constraint experiences. Factors such as education or economic background might mitigate or intensify these experiences. For example, highly educated women may encounter fewer economic constraints than less educated men, as advanced degrees often provide access to stable, high-paying occupations. Accordingly, women with greater access to social and economic capital—such as stable employment, higher education, or professional networks—may be better positioned to buffer the structural disadvantages typically associated with gender.

Nevertheless, this finding does not imply the absence of gender-based disadvantages in the Korean labor market. Previous research has documented that highly educated women still encounter wage inequality, career interruptions due to gendered expectations surrounding marriage and caregiving responsibilities (Choi et al., 2023). Youm et al. (2021) found that even within the same occupations and job levels, women consistently earned less than men, confirming the existence of a glass ceiling and gender-based rank segregation. These suggest that gender-related disadvantage is shaped by multiple intersecting factors, including educational attainment, gender role expectations, glass ceiling effects, occupational segregation, and rank segregation. Therefore, future research may benefit from adopting an intersectional approach to explore how various social factors interact with gender to influence constraint experiences.

### 4.3 Work/life outcomes of profile membership

Work volition and decent work was highest in the *privileged* group and work volition level was lowest in the *disadvantaged* group as expected. This suggests that individuals facing fewer economic constraints and marginalization are more likely to have higher work volition and secure decent work. This finding aligns with previous research that have demonstrated a strong link between constraints, work volition and decent work (Douglass et al., 2017; Duffy et al., 2019; Duffy et al., 2020). However, unexpectedly, there was no significant difference in the level of decent work among the three groups (*economically constrained*, *moderately marginalization*, and *disadvantaged* group). This may indicate that any form of constraint—whether economic or related to marginalization—can similarly impede access to decent work, with even a single constraint significantly hindering opportunities.

When decent work was decomposed into its components, safe working conditions and healthcare access were relatively high across all groups, suggesting that these components are broadly secured among Korean workers. This may be attributed to Korea’s universal healthcare system (Nam and Kim, 2019). Interestingly, the *economically constrained* group showed similar levels of safe working conditions and healthcare access as the *privileged* group. This may reflect occupational preferences among individuals with economic hardship. Previous study revealed that adolescents from lower economic backgrounds tend to view work as a means of survival, while those from higher economic backgrounds view work as a means of self-expression (Blustein et al., 2002). In addition, low-income workers place high priority on healthcare benefits for employee benefits (Danis et al., 2007). However, as this interpretation is inferential, future research should explore in more depth how decent work components are prioritized by individuals under economic hardship.

Regarding life-related outcomes, the *privileged* group reported the highest levels of life satisfaction and physical health as expected. This finding is consistent with prior research demonstrating the influence of social class on life satisfaction (Tan et al., 2020) and physical health (Adler and Ostrove, 1999). Beyond these general patterns, differences emerged among profiles with only one elevated constraint, directly addressing our research question. The *economically constrained* group exhibited lower levels of life satisfaction and physical health compared to the *moderately marginalized* group, showing similar levels to the *disadvantaged* group. This finding contrasts with Duffy et al. (2021), where the economically constrained group reported life satisfaction levels comparable to the marginalized group and significantly higher than the disadvantaged (=Marginalized and Constrained) group. In contrast, in our Korean sample, the economically constrained group showed significantly lower life satisfaction than the marginalized group, with levels similar to those of the disadvantaged group.

This result suggests that economic constraints may exert a particularly strong influence on the well-being of Koreans, potentially exceeding the impact of marginalization. Korea’s high materialistic values and collectivist orientation, where individuals are highly sensitive to context and relationships (Diener et al., 2010b; Suh, 2007), may exacerbate feelings of relative deprivation, psychological maladjustment, and frustration over unmet economic aspirations. Accordingly, targeted psychological interventions addressing relative deprivation may be essential, as further discussed in the implications section.

### 4.4 Theoretical and practical implications

Theoretically, the results support the key assumptions of the PWT, demonstrating that the two structural factors, economic constraints and marginalization, significantly influence both work and life outcomes. Furthermore, identification of profiles characterized by compounded disadvantage highlights the challenges faced by individuals experiencing constraints simultaneously. These findings underscore the need for research that examines the interactive effects of various structural constraints, rather than focusing on each in isolation. In addition, this research also expands the application of PWT within the Korean labor market. Lee et al. (2025) examined Korean workers’ profiles across five components of decent work and their relationships with demographic antecedents (e.g., gender, and educational attainment). The current findings align with theirs, indicating that individuals with lower educational backgrounds tend to report lower levels of decent work. Furthermore, this study addresses a gap in previous research by examining whether levels of decent work differ across constraint-based profiles, thereby revealing a discernible pattern of structural inequality.

Practically, these insights can inform counseling interventions for individuals facing structural constraints. Social justice-based counseling emphasizes that clients’ difficulties often arise from systemic issues such as discrimination and oppression, and effective counseling must address both individual and socio-structural factors (Fouad et al., 2006). In the context of employee counseling, it is important for counselors to assess whether economic constraints or marginalization are contributing to low perceptions of decent work and life satisfaction. If such constraints are identified, the counseling process needs to focus on helping clients understand the environmental and systemic influences on their experiences, rather than internalizing them as personal failures (O’Brien, 2001). By addressing these contextual factors, counselors can empower clients to navigate their challenges more effectively and advocate for systemic changes that mitigate the impact of structural constraints.

Specifically, this study underscore counseling strategies should aim to promote the well-being of clients experiencing economic constraints. Counseling guidelines for economically constrained individuals (Clark et al., 2020; Foss-Kelly et al., 2017) emphasize the importance of understanding how clients have coped with and survived the challenges imposed by poverty, as well as recognizing and reinforcing their strengths and resilience in the process (Clark et al., 2020; Foss-Kelly et al., 2017). Counselors also need to assist them in accessing economic resources and support and improving service accessibility that address structural barriers (Clark et al., 2020).

Finally, this study underscores the critical need for policy interventions targeting individuals experiencing economic constraints and marginalization in Korean society. Given the substantial impact of economic constraints on life satisfaction and subjective health, it is imperative to implement sustainable and effective support systems for socially vulnerable populations. In addition, attention should be directed toward individuals without a university degree. In Korea, 55% of adults have completed higher education, and approximately 70% of young adults hold higher education degrees, which is the highest rate among OECD countries (OECD, 2024). In contrast, those without higher education remain at risk of experiencing various constraints. This group takes approximately three times longer than university graduates to secure their first job, experiences higher employment rates, and typically holds lower-quality jobs (Hwang, 2021). These findings underscore the importance of tailored vocational support to enhance employment opportunities and improve the quality of life for individuals without higher education.

## 5 Limitations and conclusion

This study has several limitations. First, the use of a panel data company may have limited the sample’s representativeness. Individuals with low income or high levels of marginalization may have been underrepresented due to barriers to internet access or survey participation. This limitation is particularly relevant given that latent profile analysis is a person-centered approach, and its results can vary by sample composition. In this study, gender differences were not found across profiles, which contrasts with a substantial body of prior research documenting the marginalization and discrimination experienced by women in the Korean labor market. Future research should reexamine and expand upon these findings using more diverse and representative samples and by applying intersectional approaches that consider diverse groups of women and a range of structural and social factors. Second, the study employed a cross-sectional research design, which limits the ability to establish causal relationships between profiles and outcomes. To gain a deeper understanding of long-term changes and causality, longitudinal research is needed. Third, while we primarily focused on structural factors and their effects, we did not account for individual resources that may buffer these adverse effects. Social support and critical consciousness, for instance, could mitigate the negative impact of economic constraints on work and life outcomes (Duffy et al., 2016). Future research should incorporate such variables to provide a more nuanced understanding of the processes linking structural factors to well-being. Finally, this study did not identify the causes of marginalization, as we sought to capture the overall experience of marginalization in line with Duffy et al. (2019). However, Korea has a unique socio-cultural context such as educational background, gender, and social class. For example, *hak-beol*, a cultural notion of university prestige, may influence educational marginalization differently and should be distinguished from low educational background (Song and Lee, 2023). Future research should explore how different profiles emerge from marginalization experiences shaped by these various socio-cultural factors.

In conclusion, this study identified distinct subgroups of Korean working adults based on economic constraints and marginalization, along with their antecedents and outcomes. The findings show that individual characteristics are more strongly associated with specific profiles, while outcome differences highlight disparities in work and life experiences. These results address prior research gaps and offer meaningful implications for improving the experiences of Korean workers.

## Data Availability

The raw data supporting the conclusions of this article will be made available by the authors, without undue reservation.
